# Inferior Screw Referenced Calcar Tip Apex Distance as the Most Accurate Predictor of Mechanical Cut Out in Dual-Screw Proximal Femoral Nails

**DOI:** 10.3390/medicina62010037

**Published:** 2025-12-24

**Authors:** Yavuz Akalın, Hünkar Cagdas Bayrak, Fatih Türkmensoy, Mert Güneş, Füsun Gözen, Alpaslan Öztürk

**Affiliations:** 1 Department of Orthopedics and Traumatology, Bursa Yüksek İhtisas Training and Research Hospital, University of Health Sciences, Bursa 16310, Turkey; cagdasbayrak90@gmail.com (H.C.B.); turkmensoyfatih@gmail.com (F.T.); mertgunes10@icloud.com (M.G.); ozturkalp16@gmail.com (A.Ö.); 2 Department of Anesthesiology and Reanimation, Bursa Yüksek İhtisas Training and Research Hospital, University of Health Sciences, Bursa 16310, Turkey; fusungozen@gmail.com

**Keywords:** intertrochanteric fracture, proximal femoral nail, calcar-referenced TAD, screw cut-out, Cleveland zones, reduction quality

## Abstract

*Background and Objectives:* Screw cut-out is the most common mechanical complication after intertrochanteric fracture fixation with proximal femoral nails (PFNs). While the traditional tip–apex distance (TAD) is widely used, the calcar-referenced TAD (CalTAD) may better represent inferomedial cortical support. This study aimed to identify radiographic predictors of cut-out in dual-screw PFN fixations and establish a clinically relevant threshold for inferior-screw-based CalTAD. *Materials and Methods:* A retrospective cohort of patients treated with a dual cephalic screw PFN between 2017 and 2024 was analyzed. The implant uses two equal-diameter screws. Radiographic parameters included TAD, inferior-screw CalTAD, reduction quality, lateral wall thickness (LWT), collodiaphyseal angle (CDA), and Cleveland zone positioning. Logistic regression analyses were used to identify independent predictors of mechanical failure. *Results:* Both TAD and CalTAD values were significantly higher in patients who experienced screw cut-out. ROC analysis identified an inferior-screw-referenced CalTAD cutoff with strong predictive accuracy (AUC = 0.84). Optimal screw positioning, particularly avoiding superior placement on AP radiographs, was associated with reduced cut-out risk, while anterior positioning on the lateral view demonstrated only a borderline effect. Reduction quality showed borderline significance in univariate testing but remained independently predictive in multivariate modeling, while LWT and CDA were not significantly different between groups. *Conclusions:* Ensuring the inferior lag screw is positioned close to the calcar and achieving a low CalTAD, together with proper Cleveland zone alignment, appear to be key technical goals for minimizing mechanical cut-out in dual-screw PFN fixations. These findings support the use of inferior-screw–referenced CalTAD as a reliable and reproducible parameter for surgical optimization.

## 1. Introduction

Trochanteric femoral fractures represent one of the most common injuries encountered in orthopedic trauma practice, accounting for approximately 50% of all hip fractures [1]. Intramedullary nailing has become the fixation method of choice for unstable fracture patterns due to its superior biomechanical properties, lower surgical morbidity, and ability to facilitate early mobilization [2,3]. Despite these advantages, mechanical failure, particularly lag screw cut-out through the femoral head, remains a significant complication [4].

Since the original description by Baumgaertner et al., the Tip–Apex Distance (TAD) has been recognized as an important radiographic predictor of cut-out [5]. However, TAD was developed for single-screw sliding hip screws. It may not fully reflect the biomechanics of modern dual-screw proximal femoral nails (PFNs), in which the inferior screw provides axial and calcar support, while the superior screw primarily controls rotation [6,7]. These differences raise uncertainty regarding the most appropriate reference point for TAD assessment in dual-screw systems.

To address this limitation, several modified measurements, such as inferior-screw TAD and calcar-referenced TAD (CalTAD), have been introduced to better represent load transmission along the calcar femorale [8,9]. However, the literature remains inconclusive, and there is no established consensus regarding the optimal measurement technique for predicting cut-out in dual-screw configurations.

The purpose of this study was to investigate the predictors of cut-out and to determine the optimal TAD measurement method in patients treated with dual-screw PFN systems. We hypothesized that the inferior screw calcar-referenced TAD would demonstrate superior predictive accuracy for cut-out compared with conventional or midpoint-based TAD measurements.

## 2. Materials and Methods

This retrospective cohort study was conducted at the Department of Orthopedics and Traumatology at Bursa Yüksek Ihtisas Training and Research Hospital. The study protocol was approved by the Institutional Ethics Committee of Bursa Yüksek İhtisas Training and Research Hospital (Approval No. 2024-TBEK 2025/11-01). All procedures were performed in accordance with the principles of the Declaration of Helsinki, and institutional ethical standards were strictly observed. The study reviewed consecutive patients treated with dual-screw PFN for trochanteric femoral fractures between January 2017 and December 2024.

A retrospective database search was conducted using the International Classification of Diseases, 10th Revision (ICD-10) code S72 (femur fracture) between January 2017 and December 2024. A total of 2057 femoral fractures were identified in the institutional database. Patients aged 55 years and older who sustained low-energy domestic falls resulting in trochanteric femoral fractures classified as AO/OTA 31-A1 to A3 and were treated with a dual-screw PFN were included. Among the initially identified cases, 1083 patients with non-trochanteric fractures (e.g., femoral neck, shaft, and distal metaphysical fractures) were excluded. Additional exclusions comprised 271 patients who were lost to follow-up, deceased, or had incomplete clinical or radiographic records; and 23 patients who developed complications other than mechanical cut-out (such as implant breakage, peri-implant fracture, or deep infection requiring reoperation). 83 patients were excluded because appropriate anteroposterior and/or true lateral radiographs were unavailable, preventing reliable, reproducible radiographic measurements. Mechanical cut-out was defined as postoperative loss of reduction accompanied by lag screw cut-out or extrusion from the femoral head on serial radiographs.

**Figure 1:**
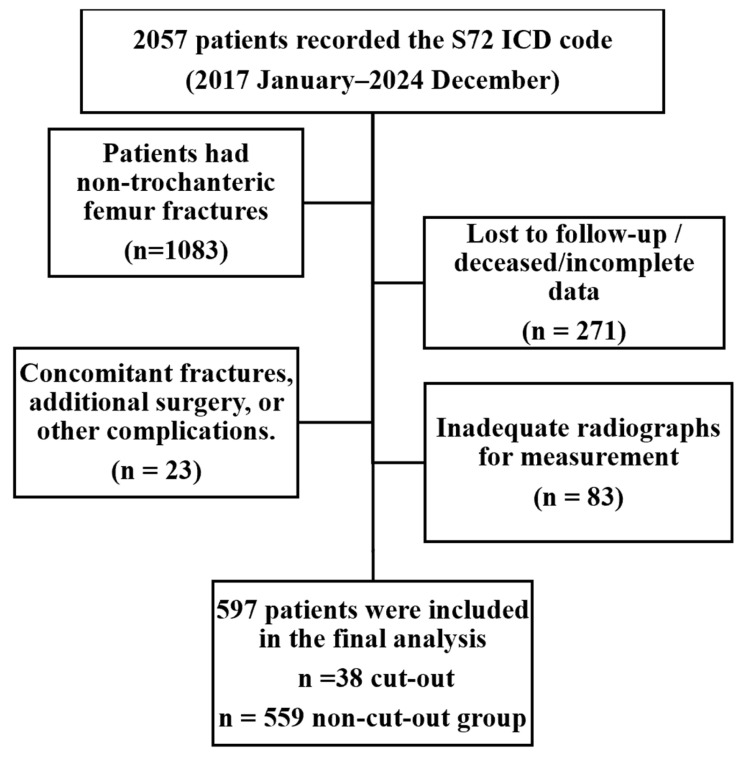
Flow diagram showing patient selection process. A total of 2057 femoral fractures were identified using the ICD-10 code *S72* between January 2017 and December 2024.

After applying these criteria, 597 patients were included in the final analysis, comprising 38 in the cut-out group and 559 in the non–cut-out group. The patient selection process is summarized in Figure 1.

All patients received standard perioperative antibiotic prophylaxis and thromboprophylaxis with low-molecular-weight heparin. Surgeries were performed in the lateral decubitus position under fluoroscopic guidance. Fracture reduction was achieved by closed manipulation; adjunctive maneuvers were used only as needed to restore alignment. Fixation was performed with a dual-screw PFN (Ortomega Proximal Femur Nail, Türkiye). Nails were inserted through a standard greater trochanteric entry, and constructs were stabilized with two cephalic screws and distal interlocking screws. Postoperative anteroposterior and lateral radiographs were obtained to verify the quality of the reduction and implant positioning. All procedures were carried out by orthopedic trauma surgeons with ≥5 years of post-training experience under senior supervision.

Patient data were obtained retrospectively from institutional medical records and radiographic archives. Demographic variables included age, sex, body mass index (BMI), fracture laterality, and American Society of Anesthesiologists (ASA) physical status classification. Clinical characteristics comprised comorbidities such as diabetes mellitus, hypertension, cardiac, pulmonary, neurological, and renal diseases. Medication history was reviewed for antiresorptive therapy and categorized as none, bisphosphonate, or denosumab treatment before trauma. Functional status was evaluated using the Modified Functional Ambulation Category (MFAC) both preoperatively and at the final follow-up. Additional clinical parameters included time to surgery (≤48 h or >48 h) and total follow-up duration (months). All data were recorded in a standardized spreadsheet template before statistical analysis.

All postoperative radiographic measurements were obtained from the hospital’s Picture Archiving and Communication System (PACS) in DICOM format to ensure measurement accuracy. Standardized radiographs were acquired within 24 h after surgery in two orthogonal planes. For anteroposterior (AP) imaging, the patient’s leg was positioned in approximately 15° of internal rotation to achieve an accurate projection of the femoral neck and head. The lateral view was taken with the contralateral limb flexed and abducted to avoid overlap, allowing clear visualization of both cephalic screws and the femoral head contour.

Fractures were classified according to the AO/OTA system as 31-A1 (simple two-part), 31-A2 (multifragmentary pertrochanteric), and 31-A3 (intertrochanteric with reverse obliquity or transverse pattern) types [10].

The femoral head was divided into nine zones using the Cleveland classification [11]. AP zones were categorized as superior, central, or inferior; lateral zones as anterior, central, or posterior. For analysis, central zones were used as the reference, and peripheral zones were evaluated separately to assess their association with cut-out.

Reduction quality was assessed on immediate postoperative radiographs using the Baumgaertner criteria [5]. Good reduction required anatomic/slight valgus alignment, <20° angulation on the lateral view, and ≤4 mm cortical displacement. Moderate reduction met alignment criteria but exceeded displacement thresholds. Poor reduction indicated >20° angulation or >4 mm displacement. These grades were used for subsequent outcome correlation.

**Midpoint-based TAD measurement**

**Figure 2:**
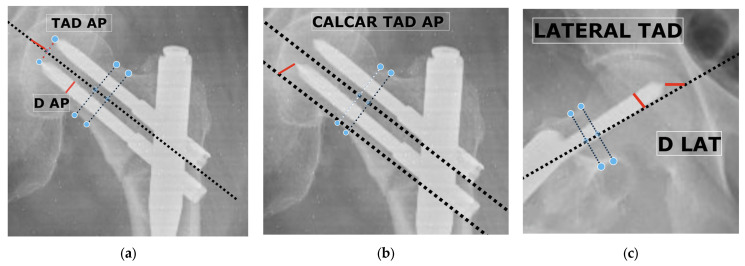
Composite illustration of midpoint-based and calcar-referenced tip–apex distance (TAD) measurement methods on postoperative radiographs. (**a**) Midpoint-based TAD is measured on the anteroposterior (AP) projection. (**b**) Calcar-referenced TAD measured on the AP projection. (**c**) Corresponding lateral projection used in both methods to calculate the total TAD (AP + lateral). Screw diameter calibration for magnification correction is demonstrated on both AP and lateral images.

Midpoint-based TAD was measured by calculating the distance from the point where the neck axis line (i.e., the line passing through the midpoint of the femoral neck) intersects the subchondral contour of the femoral head, to the midpoint between the two screw tips on both AP and lateral views; total TAD was the sum of both measurements (Figure 2a,c). This method provides an averaged estimate of screw tip depth for dual-screw constructs and has been described in previous biomechanical and clinical studies [12].

**Calcar-referenced TAD measurement**

CalTAD used the inferior screw as the reference point. Distances from the inferior screw tip to the subchondral surface were measured along the calcar trajectory on AP and parallel to the femoral neck axis on the lateral projection; the sum represented the CalTAD (Figure 2b,c). This approach aligns the measurement with the inferomedial bone stock—the region most relevant to load transfer and mechanical stability [9].

**Magnification correction**

To correct for image magnification, the following formula was applied to all distance measurements:

$$\mathrm{Corrected}\;\mathrm{distance}=(\frac{D_{\mathrm{true}}}{D_{\mathrm{AP}\;\mathrm{or}\;\mathrm{LAT}}})\times X_{\mathrm{measured}}$$

where Dtrue represents the actual diameter of the lag screw provided by the implant manufacturer, and D_AP and D_LAT are the diameters measured on AP and lateral radiographs, respectively. The final corrected TAD values were expressed in millimeters.

**Figure 3:**
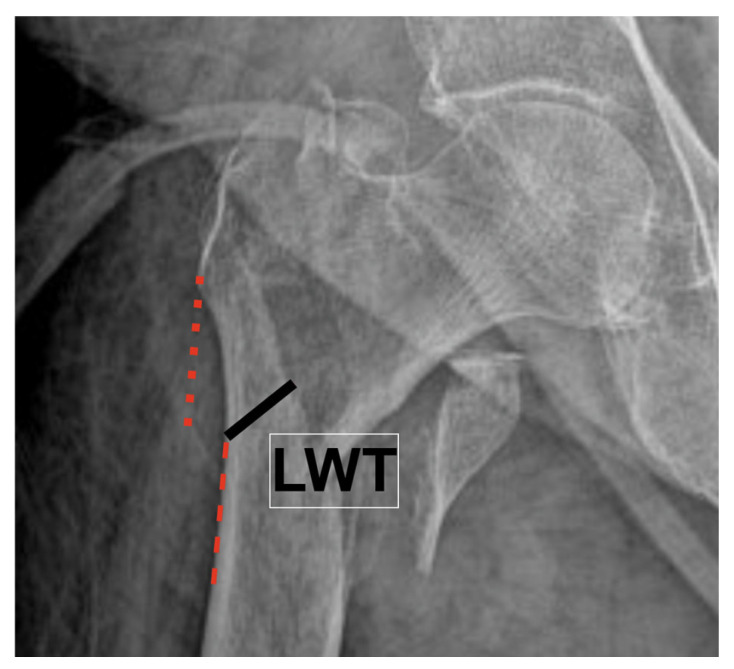
Measurement of lateral wall thickness (LWT) on postoperative anteroposterior (AP) radiograph according to the method described by Palm et al. The thickness of the lateral wall was measured along a line drawn 3 cm below the innominate tubercle of the greater trochanter at an angle of 135° to the femoral shaft axis (black line). The distance between the lateral cortex and the fracture line along this 135° line (red dotted line) represents the LWT.

Lateral wall thickness (LWT) was measured according to Palm et al. [13] along a 135° line drawn from a point 3 cm below the innominate tubercle on the AP radiograph (Figure 3).

The CDA was measured on anteroposterior radiographs to evaluate the postoperative alignment of the proximal femur. A line was drawn along the femoral shaft axis and another along the center of the femoral neck, connecting the midpoint of the femoral head to the center of the neck base.

All radiological measurements were independently performed by two orthopedic surgeons—one senior consultant and one with 10 years of experience—who were blinded to each other’s results.

**Statistical Analysis**

All statistical analyses were performed using IBM SPSS Statistics version 27.0 (IBM Corp., Armonk, NY, USA) and R version 4.5.1 (R Foundation for Statistical Computing, Vienna, Austria). The distribution of continuous variables was assessed using the Kolmogorov–Smirnov test. Since postoperative Modified Functional Ambulation Category (MFAC) scores were non-normally distributed, between-group comparisons were performed using the Mann–Whitney U test.

To evaluate potential predictors of cut-out, univariate logistic regression analyses were performed for all preoperative and intraoperative variables. Variables with *p* < 0.20 in univariate testing were selected for multivariable modeling, consistent with standard methodological recommendations. Because the number of cut-out events yielded an events-per-variable (EPV) ratio below the conventional threshold of 10, Firth’s penalized likelihood logistic regression was used in R to minimize small-sample and separation bias. In cases of multicollinearity—particularly between midpoint-based TAD and calcar-referenced TAD—correlated variables were entered into separate multivariable models to avoid coefficient distortion.

Diagnostic performance of TAD midpoint and calcar-referenced TAD values was assessed using receiver operating characteristic (ROC) analysis, and discriminative ability was quantified using the area under the ROC curve (AUC). Optimal cutoff points were identified using the Youden index (J = sensitivity + specificity − 1).

Sample size considerations were based on prior literature evaluating cut-out in dual-screw PFN constructs. Although no a priori power calculation was performed due to the nature of multivariable logistic regression, the overall cohort size and event count were sufficient for stable penalized regression modeling as recommended for low-EPV datasets.

## 3. Results

A total of 597 patients who underwent fixation with a dual-screw PFN were included, among whom 38 (6.4%) experienced mechanical cut-out. Baseline demographic and clinical characteristics are presented in Table 1. There were no significant differences between the cut-out and non–cut-out groups regarding age, BMI, ASA score, or comorbidities (all *p* > 0.05). Female sex was more frequent among patients with cut-out (78.9% vs. 64.8%, *p* = 0.075); although not statistically significant, it showed a borderline value and was therefore included in the multivariate analysis. The mean final MFAC score was significantly lower in the cut-out group (3 [1,2,3,4,5] vs. 4 [2,3,4,5], *p* < 0.001). The mean time to cut out was 6.18 ± 2.32 weeks (range, 3–11 weeks).

Operative and radiological findings are summarized in Table 2. Both TAD-midpoint and calcar-referenced TAD were significantly greater in the cut-out group (22.9 ± 4.3 mm vs. 18.3 ± 3.8 mm, and 25.4 ± 4.8 mm vs. 16.6 ± 4.1 mm, respectively; all *p* < 0.05). No significant differences were found in lateral wall thickness, collodiaphyseal angle, surgical duration, PFN type, or AO classification (all *p* > 0.05). Superior screw placement on the AP view (42.1% vs. 4.3%, *p* < 0.001) and anterior placement on the lateral view (42.1% vs. 20.6%, *p* = 0.007) were more frequent in the cut-out group. Poor reduction quality demonstrated a borderline association (*p* = 0.08) and, although not statistically significant, was included in the multivariate model due to its near-threshold value.

The results of the multivariable Firth penalized logistic regression analyses are presented in Table 3. Because the number of events per variable (EPV) was <10, penalized likelihood estimation was applied to reduce small-sample bias. Due to the strong correlation between TAD-midpoint and calcar-referenced TAD, these parameters were evaluated in two separate models to avoid multicollinearity. In Model 1, reduction quality showed an odds ratio of 1.86 (95% CI 1.09–3.14, *p* = 0.023), gender had an odds ratio of 1.61 (95% CI 0.73–3.91, *p* = 0.247), TAD-midpoint demonstrated an odds ratio of 1.10 per mm (95% CI 1.02–1.19, *p* = 0.014), superior screw position on the AP view had an odds ratio of 12.42 (95% CI 5.65–27.86, *p* < 0.001), and anterior screw position on the lateral view had an odds ratio of 1.93 (95% CI 0.96–4.44, *p* = 0.065). In Model 2, reduction quality showed an odds ratio of 1.94 (95% CI 1.10–3.44, *p* = 0.023), gender had an odds ratio of 1.48 (95% CI 0.64–3.72, *p* = 0.370), calcar-referenced TAD demonstrated an odds ratio of 1.25 per mm (95% CI 1.15–1.37, *p* < 0.001), superior screw position on the AP view had an odds ratio of 6.26 (95% CI 2.82–14.34, *p* < 0.001), and anterior screw position on the lateral view had an odds ratio of 1.89 (95% CI 0.91–4.38, *p* = 0.067).

**Figure 4:**
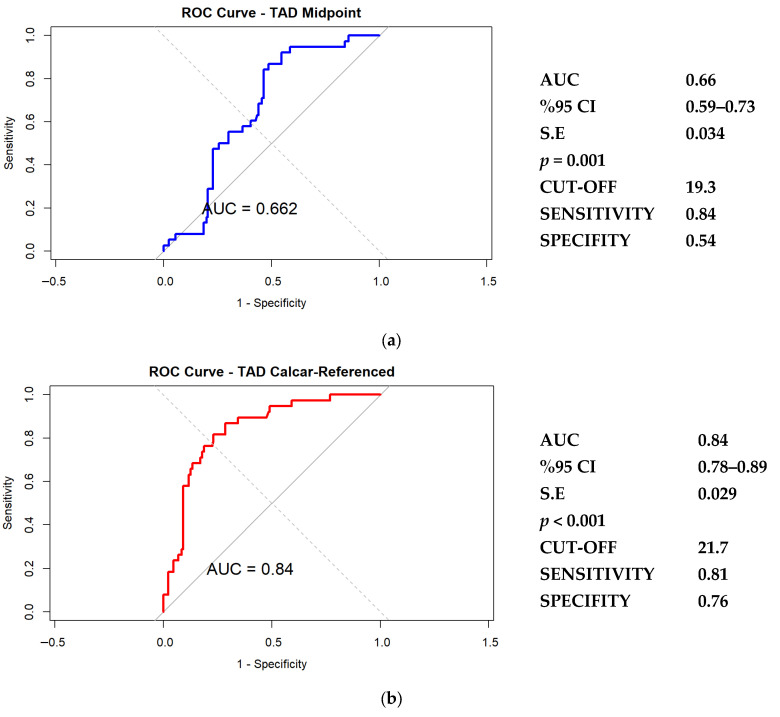
Receiver operating characteristic (ROC) curves comparing the predictive performance of midpoint-based TAD (**a**) and calcar-referenced TAD (**b**) measurements for screw cut-out.

Figure 4 illustrates the receiver operating characteristic (ROC) curves for both measurement methods. The classical midpoint-based TAD demonstrated moderate discriminative power (AUC = 0.66; optimal cutoff = 19.3 mm; sensitivity = 0.84; specificity = 0.54). The model showed good calibration (Brier score = 0.059, calibration slope ≈ 1, intercept ≈ 0), indicating that predicted probabilities closely reflected the observed risk of cut-out.

The Calcar-referenced TAD exhibited superior predictive accuracy (AUC = 0.84; cutoff = 21.7 mm; sensitivity = 0.81; specificity = 0.76), indicating better performance in identifying cases at risk of cut-out. The calcar-referenced model showed excellent agreement between predicted and observed cut-out risk (Brier ≈ 0.058; slope ≈ 1; intercept ≈ 0), supporting its clinical reliability.

The interobserver agreement for continuous variables showed excellent reliability (ICC > 0.90), while the assessment of reduction quality demonstrated a strong concordance (Cohen’s κ > 0.90).

## 4. Discussion

In the present study, TAD, screw positioning, and reduction quality emerged as the primary predictors of mechanical cut-out following dual-screw PFN fixation. These findings highlight that mechanical failure risk is largely determined by implant configuration, particularly screw placement, and by the overall quality of reduction. When the two TAD measurement techniques were compared, both midpoint based and calcar referenced values showed significant associations with cut-out. However, the calcar referenced method demonstrated higher predictive accuracy (AUC = 0.84; threshold = 21.7 mm) than the midpoint based method (AUC = 0.66; threshold = 19.3 mm). Referencing the inferior lag screw to the calcar therefore appears to provide a more anatomically relevant assessment of medial support and load transfer, especially in dual-screw constructs.

Among screw positioning parameters, superior placement on the AP view demonstrated the strongest association with mechanical failure, leading to a markedly increased risk of cut-out. This trajectory likely compromises subchondral support and alters load distribution across the head and neck junction, predisposing the construct to varus collapse even when reduction quality appears acceptable. Anterior placement on the lateral view showed a borderline effect, indicating a weaker but still relevant contribution to mechanical instability.

Although reduction quality reached statistical significance in multivariable analysis, its predictive effect was smaller compared with TAD and screw positioning. This suggests that while adequate reduction remains important, implant alignment and screw orientation exert stronger influence on mechanical outcomes in dual-screw PFN systems.

Finally, female sex demonstrated a borderline association with screw cut-out in univariate testing and was therefore included in the multivariable model. Although significance was lost after adjustment, a higher proportion of female patients was observed in the cut-out group.

Tip–apex distance (TAD) has long been recognized as a key radiographic predictor of screw cut-out in intertrochanteric fracture fixation. Baumgaertner et al. (1995) [5], first defined the concept and demonstrated a marked increase in mechanical failure when TAD exceeded 25 mm. Subsequent studies on single-screw constructs consistently confirmed the importance of maintaining short TAD values. Levine et al. [14] similarly showed increased risk above 25 mm, whereas Fujii et al. [15] reported that among multiple evaluated parameters, only TAD ≥ 20 mm was independently associated with screw cut-out.

Advancements in biomechanical understanding highlighted that centrally measured TAD does not always reflect true inferomedial support. Accordingly, the calcar-referenced TAD (CalTAD) was proposed to better represent load transfer along the calcar, which is the dominant axis of compressive force transmission. In a cohort of 571 single-screw cases, Caruso et al. [8] identified optimal thresholds of approximately 30–37 mm for TAD-based metrics, while later work by the same group demonstrated reduction in failure risk when CalTAD remained below 35 mm [16]. demonstrated that avoiding CalTAD > 35 mm reduced the likelihood of cut-out.

In dual-screw PFN systems, interpretation becomes more complex due to differing biomechanical functions of the screws. Aygün et al. [3] showed higher complication rates when TAD was >25 mm, whereas Vemparala et al. [17] noted significantly larger values in patients who experienced cut-out and reported best predictive thresholds around 30 mm. Using a midpoint-based approach, Büyükdoğan et al. [18] demonstrated that no revisions were required when TAD was ≤21.7 mm, supporting continued clinical utility of TAD even in double-screw configurations. Similar variability was also documented by Zhu et al. [19].

Recent work has adapted CalTAD to dual-screw PFN. Kyalakond et al. [9] suggested that values below 25 mm correspond with stable fixation. Our study extends this concept by evaluating CalTAD specifically using the inferior lag screw as the reference point in a system where both cephalic screws share identical geometry. This design allowed precise biomechanical interpretation, with the inferior screw representing the true calcar-supporting element and the superior screw primarily serving rotational control. Using this rationale, inferior-screw–based CalTAD demonstrated superior predictive performance (AUC = 0.84) with an optimal cutoff of approximately 21.7 mm.

Screw positioning showed a measurable influence on the risk of cut-out. Fixation was more reliable when screws were positioned within the central inferior region on the AP view, whereas superior placement aligned the screw with weaker cancellous bone and increased the likelihood of mechanical failure. In line with earlier reports [8,12,17,19], superior positioning on the AP view demonstrated the strongest association with cut-out. Anterior placement on the lateral projection had only a borderline effect, indicating a weaker and less consistent contribution to instability.

Several studies have identified reduction quality as an essential determinant of mechanical stability following intertrochanteric fracture fixation. In large clinical cohorts, Kulakoğlu et al. [20], Kinglam et al. [21], Tokgöz et al. [22], and Sukati et al. [23] reported that inadequate reduction was associated with higher rates of screw cut-out, highlighting the importance of maintaining proper alignment. In contrast, Büyükdoğan et al. [18], found no significant association in dual-screw constructs, suggesting that screw positioning and TAD-related parameters may have a stronger impact in this configuration. In our analysis, reduction quality was generally more favorable in patients without cut-out, although statistical significance was not reached in univariate testing. Because of its borderline relevance, the variable was included in multivariable modeling, where it remained an independent predictor. However, its contribution was smaller than that of screw positioning and TAD, indicating that implant-related geometric factors have a more dominant role in determining mechanical failure.

Several studies have examined the relevance of LWT when selecting fixation methods for intertrochanteric fractures. Li et al. [24] reported that reduced LWT may influence implant preference and suggested that intramedullary nails could be more suitable than dynamic hip screws in such cases. Similarly, Fahim et al. [25] noted that PFN fixation was associated with fewer lateral wall fractures and better postoperative function when preoperative LWT was below 20.5 mm. In another series, Abushahtot et al. [26] reported lower complication rates with PFN fixation in patients with reduced LWT. Although these data support PFN use in fractures with diminished wall thickness, there is limited evidence directly linking LWT to screw cut-out risk in PFN systems. In the present analysis, LWT was not associated with cut-out, suggesting that once stable fixation is obtained, variations in LWT do not appear to exert an independent effect.

The influence of CDA has also been debated. Some studies have indicated that lower CDA (<125°), corresponding to relative varus alignment, may increase mechanical load across the cephalic screws and predispose to failure after intramedullary fixation [27,28]. However, other reports found no meaningful difference between cut-out and non–cut-out groups [29]. In our cohort, CDA was similar between groups and largely above 125°, indicating that when adequate alignment and acceptable implant positioning are achieved, CDA alone does not independently contribute to cut-out risk

Sex-related differences in implant failure have been reported inconsistently. Some studies identified female sex as a risk factor for cut-out, potentially related to lower bone mineral density and thinner cortical structure [8,15]. Others, however, found no meaningful relationship after accounting for implant position and bone quality, indicating that sex alone may not determine mechanical failure [18]. In our analysis, female sex showed a borderline association in univariate testing, but this did not persist in multivariable modeling, suggesting that its influence is limited when fixation quality and bone characteristics are taken into account.

AO fracture type distribution (31-A1 to A3) was similar between groups. This supports earlier reports indicating that fracture pattern alone is not predictive of mechanical failure when fixation parameters are properly addressed [8,18].

The relevance of surgical delay to mechanical complications also remains uncertain. Although operations performed beyond 48 h have been associated with increased morbidity and mortality [30,31], these effects appear more related to systemic decline rather than implant stability. In our cohort, surgical timing did not differ meaningfully between groups, suggesting that timing alone is unlikely to affect cut-out risk when fixation quality is adequate.

This study has several important strengths. First, it includes the largest sample to date evaluating predictors of mechanical cut-out in patients treated with dual-screw PFNs that use cephalic screws of equal geometry and diameter. This uniform design allowed the inferior screw to function as a consistent reference point for CalTAD, enabling a more representative assessment of inferomedial support. The present analysis is therefore the first high-volume clinical study to examine inferior screw based CalTAD in a symmetric dual-screw configuration. Second, the study evaluated the predictive value of LWT for cut-out in PFN systems. Although LWT has been emphasized in relation to lateral wall failure and reoperation in dynamic hip screw constructs, its relevance in intramedullary devices had not been specifically addressed. The current findings indicate that when geometric stability of the construct is maintained, LWT alone does not appear to influence mechanical failure. Another strength is that all procedures were performed at a high-volume trauma center with standardized surgical practice, postoperative care and radiographic assessment, which increases internal consistency and strengthens interpretability.

This study also has limitations. First, its retrospective design limits causal inference and may introduce selection bias, although the large sample size and standardized imaging methods reduce this concern. Second, bone mineral density (BMD) data were not uniformly available. Although patients were compared according to antiresorptive treatment status and no significant differences were observed, individual DEXA measurements were incomplete. Given the advanced mean patient age and the predominance of low-energy domestic falls, osteoporosis was likely present in a substantial proportion of cases. In Turkey, based on national reimbursement criteria of the Social Security Institution (SSI), antiresorptive therapy may be initiated in high-risk patients even without DEXA testing. For this reason, treatment status was considered an appropriate surrogate for bone quality in this study. Third, implant position and alignment were evaluated using calibrated AP and lateral radiographs rather than CT based three dimensional assessment, which may not fully capture rotational deviation. Fourth, although interobserver reliability was excellent, measurement error and projection variability cannot be excluded. Finally, the cut-out incidence in this cohort was 6.4%. This rate may be slightly higher than in the broader treated population, because patients with uneventful recovery often discontinue follow-up, whereas those with symptoms are more likely to remain under surveillance. Accordingly, the observed rate should be regarded as follow-up enriched rather than population representative. Prospective studies incorporating long-term outcomes and quantitative bone evaluation will help clarify and expand these findings.

## 5. Conclusions

In conclusion, avoiding superior placement on the AP view and positioning the screw cluster toward the inferomedial region, combined with keeping CalTAD below approximately 21.7 mm, were the key positional factors associated with reduced cut-out risk in dual-screw PFN constructs with equal-diameter screws.

However, anterior positioning on the lateral projection showed only a borderline effect, whereas superior placement on the AP view was strongly associated with cut-out, indicating that coronal-plane positioning is more decisive for mechanical stability.

In our analysis, the inferior screw functioned as the primary load-bearing element, whereas the superior screw contributed mainly to rotational control. Accordingly, placing the inferior screw close to the calcar appears to improve load transfer and construct rigidity.

These findings justify further biomechanical evaluation, particularly regarding the specific contribution of inferior-screw positioning to fixation strength. The CalTAD method introduced in this study, tailored to symmetric dual-screw PFN systems, offers an anatomically grounded framework for evaluating fixation quality and may serve as a reference in future clinical and experimental investigations.

## Figures and Tables

**Table 1:** Baseline demographic and clinical characteristics of the patients with and without cut-out.

Variable	Without Cut-Out (n = 559)	With Cut-Out (n = 38)	*p*-Value	OR (95% CI)
Age (years)	80 (71–87) [79.36 ± 10.33]	81 (74–86.25) [80.53 ± 6.92]	0.729	1.01 (0.98–1.05)
BMI (kg/m^2^)	26.8 (25.3–28.3) [26.83 ± 2.02]	27.15 (25.7–28.55) [27.16 ± 2.09]	0.309	1.08 (0.92–1.27)
Preop MFAC	5 (4–6) [4.81 ± 1.36]	5 (4–6) [5.00 ± 1.25]	0.325	1.11 (0.87–1.41)
Final MFAC	4 (3–5) [3.78 ± 1.31]	3 (2–4) [2.97 ± 1.24]	<0.001 *	
Follow-up time (months)	12 (9–15) [11.97 ± 3.66]	13 (11–15) [12.76 ± 2.94]	0.223	1.06 (0.97–1.17)
Gender			0.075	
Male	197 (35.2%)	8 (21.1%)		
Female	362 (64.8%)	30 (78.9%)		2.04 (0.92–4.54)
ASA			0.223	
2–3	137 (24.5%)	6 (15.8%)		
4–5	422 (75.5%)	32 (84.2%)		1.73 (0.71–4.23)
Side			0.677	
Right	299 (53.5%)	19 (50.0%)		
Left	260 (46.5%)	19 (50.0%)		1.15 (0.60–2.22)
Before surgery > 48 h			0.708	
No	321 (57.4%)	23 (60.5%)		
Yes	238 (42.6%)	15 (39.5%)		0.88 (0.45–1.72)
Antiosteoporotic			0.440	
None	360 (64.4%)	21 (55.3%)		
Bisphosphonate	130 (23.3%)	10 (26.3%)		1.32 (0.60–2.87)
Denosumab	69 (12.3%)	7 (18.4%)		1.74 (0.71–4.25)
DM			0.938	
No	320 (57.2%)	22 (57.9%)		
Yes	239 (42.8%)	16 (42.1%)		0.97 (0.50–1.89)
HT			0.571	
No	335 (59.9%)	21 (55.3%)		
Yes	224 (40.1%)	17 (44.7%)		1.21 (0.62–2.35)
Neurological problem			0.371	
No	504 (90.2%)	32 (84.2%)		
Yes	55 (9.8%)	6 (15.8%)		1.72 (0.69–4.29)
Pulmonary problem			0.617	
No	330 (59%)	24 (63.2%)		
Yes	229 (41%)	14 (36.8%)		0.84 (0.43–1.66)
Renal disease			0.371	
No	420 (75.1%)	31 (81.6%)		
Yes	139 (24.9%)	7 (18.4%)		0.68 (0.29–1.58)
Cardiac disease			0.307	
No	383 (68.5%)	23 (60.5%)		
Yes	176 (31.5%)	15 (39.5%)		1.42 (0.72–2.79)

Values are presented as the median (25th–75th percentile) [mean ± SD] for continuous variables and as a number (percentage) for categorical variables.* Mann–Whitney U test; OR (95% CI) and *p*-values from univariate logistic regression. ASA: American Society of Anesthesiologists; MFAC: Modified Functional Ambulation Category; BMI: Body mass index.

**Table 2:** Intraoperative parameters, radiological measurements, and Cleveland zone distribution.

Variable	Without Cut-Out (n = 559)	With Cut-Out (n = 38)	*p*-Value	OR (95% CI)
Lateral wall thickness (mm)	16.68 (12.3–22.32) [17.87 ± 7.66]	16.98 (13.16–21.53) [17.55 ± 6.41]	0.799	0.99 (0.95–1.04)
TAD midpoint (mm)	18.32 (15.27–23.19) [19.92 ± 6.17]	22.86 (19.88–25.14) [22.86 ± 5.06]	0.005	1.07 (1.02–1.12)
Calcar-referenced TAD (mm)	16.61 (13.77–21.70) [17.57 ± 5.58]	25.38 (22.88–27.54) [25.01 ± 4.96]	<0.001	1.24 (1.16–1.32)
CDA (°)	129.3 (127.3–132.5) [130.08 ± 4.36]	129.15 (126.85–133.68) [130.38 ± 4.75]	0.686	1.02 (0.94–1.09)
Surgery duration (min)	60 (55–70) [61.49 ± 9.42]	60 (53.75–65) [58.55 ± 8.21]	0.230	0.98 (0.94–1.01)
PFN type			0.269	
Short	536 (95.9%)	35 (92.1%)		
Long	23 (4.1%)	3 (7.9%)		—
AO Classification			0.753	
31A1 (Ref)	198 (35.4%)	12 (31.6%)		
31A2 vs. 31A1	319 (57.1%)	22 (57.9%)	0.727	1.14 (0.55–2.35)
31A3 vs. 31A1	42 (7.5%)	4 (10.5%)	0.453	1.57 (0.48–5.11)
Reduction quality			0.080	
Good (Ref)	375 (67.1%)	19 (50%)		
Moderate vs. Good	139 (24.9%)	13 (34.2%)	0.101	1.85 (0.89–3.84)
Poor vs. Good	45 (8.1%)	6 (15.8%)	0.050	2.63 (1.00–6.93)
Cleveland AP			<0.001	
Inferior (Ref)	64 (11.4%)	1 (2.6%)		
Medium vs. Inferior	471 (84.3%)	21 (55.3%)	0.310	2.85 (0.38–21.58)
Superior vs. Inferior	24 (4.3%)	16 (42.1%)	<0.001	42.67 (5.36–339.49)
Cleveland LAT			0.003	
Posterior + Medium (Ref)	444 (79.4%)	22 (57.9%)		
Anterior	115 (20.6%)	16 (42.1%)	0.003	2.42 (1.34–4.38)

Values are presented as the median (25th–75th percentile) [mean ± SD] for continuous variables and as a number (percentage) for categorical variables. Monovariate logistic regression analysis applied. TAD: Tip-Apex Distance; CDA: Caput-collum-diaphyseal angle; PFN: Proximal femoral nail.

**Table 3:** Firth’s Penalized Logistic Regression Analysis.

Variable	*p* Value	OR (95% CI)	*p* Value	OR (95% CI)
Regression Models	Model 1		Model 2	
Reduction quality	0.023	1.86 (1.09–3.14)	0.023	1.94 (1.10–3.44)
Gender (Female)	0.247	1.61 (0.73–3.91)	0.370	1.48 (0.64–3.72)
TAD-midpoint (per mm)	0.014	1.10 (1.02–1.19)	*	*
TAD-calcar-referenced (per mm)	*	*	<0.001	1.25 (1.15–1.37)
Cleveland AP (Superior position)	<0.001	12.42 (5.65–27.86)	<0.001	6.26 (2.82–14.34)
Cleveland Lateral (Anterior position)	0.065	1.93 (0.96–4.44)	0.067	1.89 (0.91–4.38)

Due to a limited number of cut-out events (EPV < 10), Firth’s penalized likelihood logistic regression was used to minimize small-sample bias. Because of the strong correlation between TAD-midpoint and calcar-referenced TAD (r = –0.79), these variables were analyzed in separate penalized models to avoid multicollinearity and preserve model stability. * = excluded models.

## Data Availability

The data presented in this study are available on reasonable request from the corresponding author. The data are not publicly available due to institutional privacy regulations.

## Abbreviations

The following abbreviations are used in this manuscript:

**Abbreviation**	**Definition**
PFN	Proximal Femoral Nail
TAD	Tip–Apex Distance
CalTAD	Calcar-Referenced Tip–Apex Distance
CDA	Caput–Collum–Diaphyseal Angle
LWT	Lateral Wall Thickness
MFAC	Modified Functional Ambulation Category
ROC	Receiver Operating Characteristic
AUC	Area Under the Curve
ASA	American Society of Anesthesiologists
BMD	Bone Mineral Density
DICOM	Digital Imaging and Communications in Medicine
